# Neuro-Behçet Disease Presenting as a Bulbar Lesion: A Case Report

**DOI:** 10.7759/cureus.68236

**Published:** 2024-08-30

**Authors:** Mariana Certal, Ana Sofia Alves, Marta B Santos, Fernando Salvador, Michel Mendes

**Affiliations:** 1 Internal Medicine, Unidade Local de Saúde (ULS) de Trás-os-Montes e Alto Douro, Chaves, PRT; 2 Internal Medicine, Unidade Local de Saúde (ULS) de Trás-os-Montes e Alto Douro, Vila Real, PRT; 3 Neurology, Unidade Local de Saúde (ULS) de Trás-os-Montes e Alto Douro, Vila Real, PRT

**Keywords:** immune inflammatory disease, wallenberg’s syndrome, nervous system, neurobehçet, behçet’s disease

## Abstract

Behçet's disease (BD), also called neuro-Behçet (NB), is a multisystem inflammatory disease that can affect the nervous system. The authors hereby present the case of a 46-year-old woman with a previous diagnosis of BD with cutaneous, articular, and ocular involvement. The patient was admitted to the emergency room with intense posterior cervical pain, right eyelid ptosis accompanied by anisocoria, left arm hemiparesis, left hemihypoesthesia, right dysmetria, and postural instability. A cerebral MRI revealed a right laterobulbar oval T2 hyperintense lesion, suggesting a diagnosis of NB.

Treatment with methylprednisolone pulses and, later, azathioprine was then started. The patient showed progressive improvement in her clinical condition and an imagological resolution of the bulbar lesion. This case highlights the importance of an accurate diagnosis - based on clinical history and imaging studies - for an early initiation of the specific therapy.

## Introduction

Behçet's disease (BD) is a multisystemic inflammatory vascular disease of unknown origin. Its clinical triad includes recurrent oral, and/or genital ulcers and uveitis. However, BD can also affect the eyes, skin, mucous, membranes, vascular system (primarily the venous system), lungs, gastrointestinal tract, and central nervous system [1].

Epidemiologically, BD mainly affects adults between 20 and 40 years old. The natural course of the disease is characterized by periods of increased activity and remission. In a study by Kural-Seyahi et al., complete remission was observed in approximately 60% of patients during 20 years of follow-up [2].

The etiopathogenesis of BD is unknown, but it is believed to be related to genetic and environmental factors, such as infectious agents. Several genetic studies have confirmed that HLA-B51 is the strongest genetic susceptibility factor. Additionally, new non-HLA susceptibility genes have also been identified [3].

Involvement of the nervous system, known as neuro-Behçet (NB), occurs in 3%-30% of all cases. It can be characterized by symptoms and signs secondary to parenchymal inflammatory lesions, venous sinus thrombosis, or recurrent meningoencephalitis [4,5]. Histopathological findings suggest a vasculitis involvement in some cases, while others show low-grade chronic nonspecific inflammation [6]. Clinical and imaging evidence suggests that primary neurological involvement in BD can be subclassified into two main forms. (1) Parenchymal (more prevalent) is characterized by a vascular-inflammatory disease of the central nervous system with subacute, focal, or multifocal parenchymal involvement. Brainstem involvement is the most common presentation. (2) Non-parenchymal includes cerebral venous sinus thrombosis and secondary intracranial hypertension, or more rarely, arterial involvement. This form occurs in 10%-20% of cases and typically presents a better neurological outcome.

Both phenotypes are rarely found in the same patient, suggesting that their pathophysiology is likely different [7]. Isolated behavioral syndromes and peripheral nervous system involvement are uncommon. Secondary neurological changes due to the systemic involvement of BD, such as cerebral thrombosis resulting from cardiac complications, as well as neurological manifestations associated with BD treatment, are considered secondary neurological involvement. Since neurological involvement in this disease is very heterogeneous, it is difficult to predict its course, the prognosis, and the response to treatment. Therefore, this article aims to present a clinical case of NB to promote earlier initiation of targeted therapy, emphasizing the importance of considering this pathology as a differential diagnosis in the context of acute neurological deficits.

This clinical case was previously presented as a meeting abstract at the VII National Congress of Autoimmunity and XXVII Annual Meeting of NEDAI in Portugal.

## Case presentation

A 46-year-old female was admitted to the emergency room with posterior cervical pain rated 8/10 in intensity. She also reported blurred vision and nausea. On admission, she was in good general condition, apyretic, acyanotic, and eupneic, with a blood pressure of 180/103 mmHg, normocardiac, and without alterations on cardiac or pulmonary auscultation. Neurological examination revealed right-sided Horner’s syndrome, normal facial sensitivity, facial symmetry, loss of the gag reflex on the right side, preserved and symmetrical muscle strength, left-sided thermoalgesic and tactile hemi-hypoesthesia, and right-sided ataxia, consistent with Wallenberg syndrome.

Her medical history included essential hypertension and dyslipidemia (brought under control by medication), BD diagnosed nine years ago with mucocutaneous involvement (pseudofolliculitis, erythema nodosum, recurrent oral and genital aphthosis), articular and ocular involvement (posterior uveitis). She had a previous venous thrombosis in the right eye and is a smoker with a history of four units-pack-years. Her current medications included methotrexate 17.5 mg weekly for one year, supplemented with 10 mg of folic acid weekly, and colchicine 1 mg/day. There was no personal or family history of thrombotic events.

As part of the investigation, an analytical study was conducted (Table 1), along with an electrocardiogram, and a cerebral computed tomography (CT) with an angiographic study. The CT did not reveal any pathological alterations.

**Table 1:** Analytical study on admission. ACE - Angiotensin-converting enzyme; ANA - Antinuclear antibodies; CK - Creatinine kinase; CK-MB - Creatinine kinase-MB; CMV - Cytomegalovirus; CRP - C-reactive protein; ESR - Erythrocyte sedimentation rate; HCV - Hepatitis C virus; HIV - Human immunodeficiency virus; Ig - Immunoglobulin; INR - International normalized ratio; RPR - Rapid plasma reagin test; TPHA/TPPA/TP - Treponema pallidum hemagglutination assay/particle agglutination assay.

Parameter	Result	Reference range	Parameter	Result
Hemoglobin	16.3	12 - 16 g/dL	Anti-CMV	
Leukocytes	10,050	4,000 - 11,000/µL	IgG	Reactive
Platelets	276,000	150,000 - 400,000/µL	IgM	Non-reactive
ESR	9	0 - 15 mm/1ªh	HBs antigen	Negative
CRP	0.13	< 0.5 mg/L	Anti-Hbs	Non-reactive
Creatinine	0.6	0.6 - 1.1 mg/dL	Anti-HCV	Non-reactive
Sodium	140	135 - 147 mEq/L	Anti-HIV	Non-reactive
Potassium	3.4	3.7 - 5.1 mEq/L	ANA	Negative
Calcium	8.8	8.6 - 10 mg/dL	Anti-β2 glycoprotein	
CK	95	< 170 U/L	IgG	Negative
CK-MB	< 25	< 25 U/L	IgM	Negative
Troponin T	0.008	< 0.05 ng/mL	Anti-cardiolipin	
Myoglobin	< 21	< 58 ng/mL	IgG	Negative
INR	1.04	< 1.2	IgM	Negative
d-dimers	0.42	0.0 - 0.5 µg/mL	TPHA/TPPA/TP	Negative
ACE	26	20 - 70 U/L	RPR	Negative

She was hospitalized for surveillance and further investigation. The lumbar puncture results showed normal biochemical and cytological parameters, no microbiological isolations, and the presence of two bands of oligoclonal characteristics in the cerebrospinal fluid (with correspondence in the serum), indicating altered blood-brain barrier permeability (Table 2). 

**Table 2:** Characteristics of cerebrospinal fluid. Ig - Immunoglobulin; LDH - Lactate dehydrogenase; TPHA/TPPA/TP - Treponema pallidum hemagglutination assay/particle agglutination assay; VDRL - Venereal disease research laboratory.

Parameter	Result	Reference range	Parameter	Result
Cells	0	-	Oligoclonal bands	Positives
Erythrocytes	150	-	VDRL	Negative
Glucose	57	40 - 76 mg/dL	TPHA/TPPA/TP	Negative
Total proteins	0.54	< 0.45 g/L		
LDH	25	< 35 U/L		
IgG	3.13 mg/dL	< 3.6 mg/dL		

**Figure 1:**
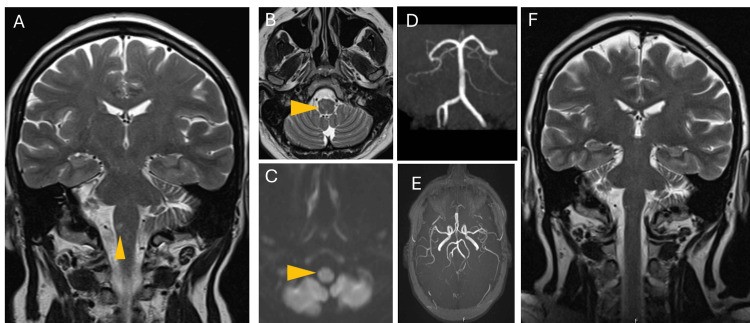
Brain MRI. T2-weighted in coronal (A) and axial (B) slices revealing a hyperintense lesion, right laterobulbar, and slightly hyperintense on DWI sequence (C). MRA without signs suggestive of dissection or vasculitis (D, E). Subsequent coronal slice showing a shrunken bulbar lesion on T2 weighting (F).

Magnetic resonance imaging (MRI) revealed an oval lesion hyperintense on T2 and slightly hyperintense on diffusion imaging, located in the right laterobulbar region, without abnormal contrast uptake or alterations in the vascular tree suggestive of vasculitis, raising hypotheses of inflammatory lesion or lacunar infarct (Figures 1A-1F).

In this context, the diagnosis of NB with parenchymal involvement was assumed - symptomatically corresponding to the Wallenberg syndrome. Treatment was started with methylprednisolone pulses 1 g for five days, followed by prednisolone (PDN) 1 mg/kg/day (maximum dose of 60 mg) with subsequent progressive tapering. The therapy was adjusted by adding adjunctive immunosuppression with azathioprine, titrated to a dose of 2.5 mg/kg/day, according to the literature review and “2018 update of the EULAR recommendations for the management of Behçet’s syndrome” [8]. Prophylaxis with calcium, vitamin D, and trimethoprim + sulfamethoxazole was started. Daily physiotherapy rehabilitation was maintained, and she was followed up in the autoimmune diseases internal medicine clinic.

The patient showed progressive clinical improvement of the neurological deficits, with recovery of her autonomy. Ten months after starting therapy, a new MRI showed complete resolution of the bulbar lesion and no relapses of disease with tapering PDN (Figure 1D).

## Discussion

The prevalence of central nervous system involvement in patients with BD is significant. Neurological symptoms typically manifest, on average, three to six years after the onset of BD, usually between the third and fourth decades of life. However, some patients may develop neurological involvement simultaneously with or even before the onset of BD [4].

The non-specific nature of neurological symptoms associated with BD necessitates a high level of clinical suspicion. In this case, the symptoms appeared acutely, nine years after the initial diagnosis of BD, presenting as Wallenberg syndrome.

In the approach of an acute focal neurological deficit, the association between the clinical history and the imaging findings is extremely important in the attempt to establish the correct diagnosis and initiate therapy, as soon as possible. In this instance, despite the patient having vascular risk factors such as hypertension, dyslipidemia, and smoking, CT did not reveal any signs of arterial occlusion or dissection. However, brainstem involvement is the most common presentation of BD-related neurological involvement, and MRI showed a right-sided laterobulbar lesion with imaging features atypical for vascular injury but consistent with parenchymal BD. Contrary to what is typically observed, the cerebrospinal fluid did not show pleocytosis or elevated protein levels. Nonetheless, oligoclonal bands corresponding to those found in the serum were detected, a finding that, while rare in BD, has been previously reported [9]. Based on clinical and imaging findings, and after excluding other differential diagnoses, a diagnosis of BD flare in NB form was established. The patient received pulses of methylprednisolone at 1 g/day for five days, and adjuvant immunosuppression was switched from methotrexate to azathioprine. Following the initiation of immunosuppressive therapy, the patient showed significant clinical improvement, supporting an inflammatory etiology. She was discharged with outpatient therapy of Azathioprine and a tapering dose of PDN. The efficacy of this treatment was monitored through clinical evolution and imaging to confirm the fading of the lesion.

## Conclusions

This case highlights the importance of promptly addressing acute neurological deficits, the integration with clinical history, and the correlation with imaging findings for accurate diagnosis and initiation of specific therapy. Treatment with high doses of intravenous methylprednisolone, followed by a transition to oral treatment with gradual tapering, along with adjuvant immunosuppression using azathioprine, allowed for a favorable patient outcome.

NB remains a challenging diagnosis to establish and exclude due to the presence of numerous other more prevalent diseases and conditions with similar presentations. Diagnosis is based on clinical presentation and typical MRI lesions, which commonly disappear with specific disease treatment.
